# Neckio: Motivating Neck Exercises in Computer Workers

**DOI:** 10.3390/s20174928

**Published:** 2020-08-31

**Authors:** Panos Markopoulos, Xiaoyu Shen, Qi Wang, Annick Timmermans

**Affiliations:** 1 Department of Industrial Design, Eindhoven University of Technology, 5612 AZ Eindhoven, The Netherlands; shenxiaoyu0220@gmail.com; 2 College of Design & Innovation at Tongji University, Shanghai 200093, China; qiwangdesign@tongji.edu.cn; 3 Department of Rehabilitation Sciences, REVAL, University of Hasselt, 4000 Limburg, Belgium; annick.timmermans@uhasselt.be

**Keywords:** interaction design, neck health, neck posture, therapeutic exercise, work environment, behavior change support technology

## Abstract

Neck pain is common among computer workers who may spend too much time in a static posture facing their display. Regular breaks and variety in one’s posture can help to prevent discomfort and pain. In order to understand how to support computer workers to do so regularly, we surveyed a convenience sample of computer workers (N = 130) regarding their work habits and their attitudes towards neck exercises at the workplace. The survey showed that they are highly motivated, but not able to comply with a neck exercise program. To address this challenge, we designed Neckio, a system that is aimed at encouraging posture variation and facilitating neck exercises at work. Neckio consists in an interactive application and a wireless angulation sensing appliance that can be mounted on the headset that office workers often use for reasons of privacy. Next to providing an interactive exercise program suitable for the workplace, its design places emphasis on an engaging user experience. We report a short-term user experience valuation of Neckio in an actual office environment (N = 10). Participants rated the overall user experience positively and reported to be intrinsically motivated to do the neck exercises. These results indicate the potential of the Neckio as a behavior change support technology to reduce the risk of developing neck pain in computer workers.

## 1. Introduction

Neck pain is a common complaint in industrialized countries and constitutes a major medical and socioeconomic problem. It is one of the three most commonly reported complaints of the musculoskeletal system: Population based studies [1,2,3,4,5] suggest a lifetime prevalence of 67–70% and a point prevalence of between 10–12%. A cross sectional study found that the one-year prevalence of neck pain among office workers was 45.5% [6].

Work-related neck disorders are common in office workers [6]. However, also 60% of persons between the ages of 10 and 17 experience discomfort in the neck region during computer use and an association has been established between the number of hours of computer use and general musculoskeletal problems [7,8] and neck pain specifically [9]. Hakala et al. [10] report that daily use of computers for more than 2–3 h is associated with neck shoulder pain and more than 5 h with low back pain in individuals aged 14–18. In adults, working for more than 15 h per week with computer screens has been shown to increase the risk for developing neck shoulder problems [11].

Cagnie et al. [6] argue that work factors contributing to neck shoulder pain may be physical, such as prolonged flexed neck posture, prolonged sedentary time, and repetitive movement, psychosocial, such as mental tiredness and shortage of personnel, as well as individual variables, such as age, gender, etc. Importantly, they note how being physically active decreases the likelihood of having neck pain. Similar association patterns are reported by Smith et al. [12] who argue that there is an association between neck pain and long hours of computer-related work. Computers allow users to accomplish their tasks with little or no regular large movements. Such absence of posture variation has been recognized as a health risk [13]. These findings indicate the need for stiumlating posture variation in computer workers to contribute towards a positive psychosocial work environment.

An early study by Anderson et al. [14] found that a one year physical exercise intervention, comprising of specific neck shoulder resistance training or all-round physical exercise can decrease neck pain in office workers. This result is supported by a more recent systematic review [15], which argues that strengthening exercises of musculature in the neck and shoulder area helps reduce neck pain in office workers. Recent research has also demonstrated the preventive value of exercises for work-related neck pain. Sihawong et al. [16] report that a 12 month exercise program in office workers, comprising of stretching (30 s, twice per day) and neck flexor endurance training (10 times, twice a week) reduced the incidence of neck pain in office workers by 55%. Tunwattanapong et al. [17] show that a regular stretching exercise program (2 × 15 min per day, 20 to 30 repetitions, more than 3 times per week, and for a minimum of 4 weeks) can decrease neck pain and improve neck function and quality of life of office workers. This exercise program comprised of neck stretching, shoulder stretching, shoulder rolling, trunk stretching, and showed positive effects in just four weeks. Besides, the protective effect of rest breaks has been observed in several studies [6,18,19,20]. Breaks allow for a reduction in computer exposure, and more especially enable posture variation and muscle relaxation. A field study [21] of sitting posture and sedentary time in the office reports that workers on average spend a long time seated without breaks, but also that with appropriate reminders to take short breaks every hour, high compliance can be achieved. This finding leads to the question of how behavior change interventions targeting neck pain can be supported by interactive technology.

At present, products that address neck pain prevention can be divided into two categories: posture-tracking devices and exercise-coaching applications. Posture-tracking devices can be wearable, such as experimental devices that use flex sensors [22,23,24] and the commercial system Alex [25]. Alternatively, they can be off the body sensor-based devices, such as Measuring Chair [21] or camera-based systems [26] that track posture at a fixed location, reminding users to sit upright and providing feedback on their posture. Exercise coaching applications guide users in carrying out physical exercises with media such as pictures, video or animation or, virtual reality (e.g., see [27]).

Unsupervised physical exercise relies on the users being aware of their own posture and being self-disciplined enough to adhere to the exercise program. The latter is particularly challenging for prevention, which by its nature relies on long term adherence. These two challenges can be addressed through a combination of self-tracking and coaching applications.

This paper describes the design of Neckio, a system that is intended to help computer users to perform personally scheduled neck exercises in an interactive and motivating way. We followed an iterative user-centred design approach to investigate what kind of interactive application can increase users’ adherence to regular neck exercises and can motivate them to change sedentary behaviors that affect their neck health. Our design process started with a survey of computer workers, which found that, in order for this system to be used during work hours, it should be minimally intrusive and fit well into the social and physical context of a modern workplace, and should be possible to use flexibly at the various locations modern office workers (e.g., flex workers) find themselves.

**Figure 1:**
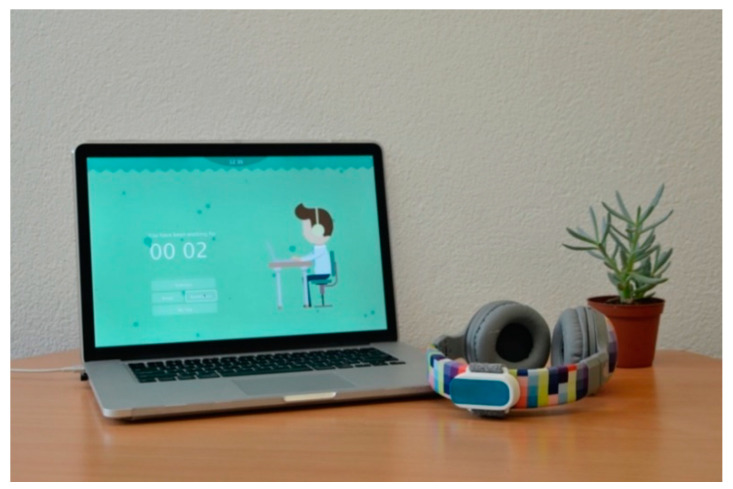
Project result, Neckio, a smart system comprises a desktop application and a headphone-attached tracking band.

The Neckio system presented below monitors computer usage and neck prolonged static posture in order to remind users to take short rest breaks and perform a series of stretches for their neck. The exercises can be done discretely in cases of a shared office environment. The system (see Figure 1) includes a small sensing contraption that is mounted on the user’s headphones, which are commonly worn at shared office environments and does not require the user to carry bulky extra devices such as VR goggles which could potentially attract negative attention. The user test results that are presented in the following sections illustrate how Neckio provides a positive user experience that can motivate users to take breaks regularly and perform the advised neck exercises. Before reporting on the system and its evaluation, we motivate the design approach and the user studies that informed the design of Neckio.

## 2. The Design of Neckio

Our design process followed the Double Diamond model by the Design Council [28]. This model prescribes an iterative process, allowing for iteration and the testing of ideas with stakeholders, which structures the design process along four major phases: Discovery and Definition, which result in a design brief, and Development and Delivery, which pertain to developing, evaluating, and documenting a design concept.

Initial consultations with physiotherapists and clinical researchers brought home the message that rather than striving to sustain an optimal posture through self-tracking, regular breaks and stretching exercises can help prevent neck pain in computer users. Moreover, they argued that while such exercises do not require exertion, doing them regularly could be a challenge for computer workers. We thus decided to go beyond using sensors to track posture and to provide notification alerts for poor posture as is done with some commercial systems (e.g., Alex [25]) or other experimental posture-tracking applications (e.g., [29,30]). Such posture tracking could be helpful, but suffers from an important limitation: people’s neck posture is most likely to be static for a long time exactly when they need to work long hours and are under time pressure, in which case they are also likely to ignore posture related notifications.

Such insights provided the input to a co-creation session involving four interaction designers, which identified four general design directions that appeared promising:

- Creating awareness of neck posture by providing peripheral information displays (meaning that they should not require user’s focus of attention [31]).
- Creating intuitive interactions to relate cues to desired body movements.
- Guiding exercises with music.
- Tracking posture with the laptop’s camera or with a wearable sensor.

Following these initial explorations, we decided to approach our design challenge as designing a system to support behavior change in computer users and a user survey was carried out in order to unpack the challenge accordingly.

### 2.1. Survey of Computer Users

An online survey was carried out in order to understand the attitudes computer users hold towards neck exercises, and more specifically what hinders their adherence to regular neck exercises. A survey approach was considered preferable in order to cast a wide net and engage with numerous informants in order to profile the potential users of Neckio.

#### 2.1.1. Method

A convenience sample was recruited through social media. The inclusion criteria were: to be working more than six hours daily on a computer. Survey questions were inspired from the Fogg Behavior Model (FBM) [32], which stipulates that, for a person to perform a target behavior, they must be:

- Sufficiently motivated.
- Have the ability to perform the behavior.
- Be triggered to perform the behavior.

The survey items were scored on a 5-point Likert scale regarding the respondent’s ability to carry out exercise in terms of availability of time, physical effort, cognitive effort, social deviance and the influence of routines, and their motivation to do so, in terms of their prior efforts to do so and their attitudes regarding the value of neck exercises in preventing neck pain.

#### 2.1.2. Results

In total, 130 adults participated in this online survey (56.4% male, and 44.6% female). Despite the fact that this was quite a young sample (47.7% between 18–30 and 23.9% between 31–40), 84% of them had already experienced different levels of neck pain in the 12 months prior to the study (47% early; 11% mid; 26% severe). They reported spending on working days an average of 7.59 h (SD = 1.73) seated, while it is known that working with a computer for more than 6 h per day is associated with work-related upper limb disorders in all body regions [33]. A total of 75% of the respondents work in an open space, e.g., open-plan offices, library, etc., which could hinder their ability to perform neck exercises.

Our survey participants were highly motivated by the hope of preventing or reducing neck pain. In total, 90% of them knew that exercise can reduce neck pain, and 64.1% of them have tried such exercises before.

In terms of the Fogg Behavioral Model [32], we could argue that such office workers have a sufficiently high motivation to do exercises that can help prevent neck pain. On the contrary, their ability to carry out exercises is relatively low. Only 5.3% reported to have kept to the exercise program for more than a week. On a scale from 1 (easy) to 5 (difficult), participants rated as most difficult the challenge of finding the time to carry out neck exercises (M = 3, STD = 0.97), the cognitive effort these require, i.e., remembering how to do the exercises M = (2.73, STD = 1.22), overcoming social deviance i.e., feeling awkward to be seen doing the exercises in public, M = 2.85, STD = 1.31), and diverting from their routine (M = 3.83, STD = 1.24). Surprisingly for us, they considered the exercises as such easy to do (M = 0.84, STD = 0.98).

Based on the survey, the user group was narrowed down to computer workers, spending more than 6 h per day in sedentary working in open office spaces, who have neck pain history; although, people who have neck pain caused by physical injury or damage are not included here. Following the model by Fogg [32], since our target users reported a high motivation and low ability, the designed system should support short exercises breaks to reduce the demand on time and provide a trigger which can serve as a reminder to the exercises and as a facilitator to help workers in doing them. Especially for open office workers, the exercises should feel easy to do in the presence of others.

## 3. The Design of Neckio

Following the insights gained through the discovery phase of the double diamond process [28], we set out with the design brief of creating a system that:

- Reminds workers to do neck exercises and take regular rest breaks.
- Guides users to do exercises.
- Provides meaningful feedback to users about the variation in posture and performance on exercises.
- Monitors users in a minimally intrusive way and fits into their daily work context.
- Encourage users to schedule exercises at suitable times and make commitments to it.

The next phase of the design process was structured in two main iterations described briefly below.

### 3.1. Design Iteration 1

Our first design iteration examined the feasibility of tracking posture unobtrusively. The option of installing sensors on furniture was considered but it bounds the solution to a fixed location, which cannot be assumed in flex office environments or nomadic workers. The need for ubiquity could be addressed by using computer vision for head posture tracking and specifically by using the camera, which is embedded on laptops or computer monitors. However, currently available computer vision applications do not yet track movements such as rotation, extension and flexion reliably and can be sensitive to environmental lighting variations.

Noticing that it is common amongst open space workers to wear headphones in order to isolate themselves from environmental distractions, and given that these people are high-risk individuals for developing neck pain, we saw the potential of enhancing headsets with sensors in order to track neck movements and approximate sitting posture. Unlike the Alex system [25], which has sensors placed on the nape of the neck, mounting the sensors for tracking neck posture on a headset allows the wearer to move and exercise freely. The headphones themselves can be used to deliver reminders or to guide the user through audio feedback.

**Figure 2:**
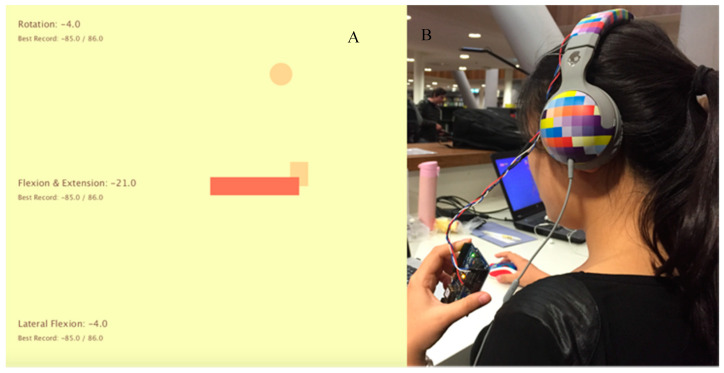
Exercise feedback exploration. (**a**) Real-time angulation data and the maximal angle reached are displayed on left side in degrees. The lengths of two bars represent, respectively, the tilt angle during Lateral bending (X-axis) and flexion and extension (Y-axis). A circle moves on a circular pathway whose position indicates the rotation angle during neck rotation (Z-axis). (**b**) The sensor is placed on the headphone and connected to Arduino by wire.

A feasibility prototype was developed using an Arduino microcontroller and the InvenSense MPU6050 sensor which contains an MEMS accelerometer and a MEMS gyroscope. In this initial feasibility prototype the data is sent via a USB cable to a laptop where the angulation of head movements is visualized using Processing with simple graphics (see Figure 2).

As Figure 2 shows, the lengths of the two bars represent the tilt angle during lateral bending (X-axis) and during flexion and extension (Y-axis). A circular puck moves along a half-circle pathway to illustrate the neck rotation angle (Z-axis). In order to support the self-assessment of neck flexibility, the color of the bars and the circle change when the user reaches the targeted range of motion for a particular exercise. The real-time angulation data together with the largest angle the user has reached in one exercise are displayed visually.

Formative and unstructured user tests were carried out to guide the next design iteration. While further improvements were still necessary, the user interface was considered to provide a clear visualization the of user’s exercise performance. To further develop this concept prototype, we found it most useful to add exercise guidance as feed-forward [34] to indicate the required movements, speed and range of motion so that people who are unfamiliar with the neck exercises can be assisted. From the perspective of product design, the USB cable connection used in this prototype was found to be uncomfortable and unappealing to wear. So, for the next iteration, we set out to make data transmission wireless and to reduce the size of the prototype.

### 3.2. Design Iteration 2

The aim of the improved prototype was to create a system that can be used by computer users during daily computer work and motivate regular neck exercise and rest breaks in the long term. The second iteration focused on improving the user experience and the product design. The final prototype Neckio (see Figure 1) comprises a software program and a wireless tracking device on a headphone. The exercise content was developed in consultation with physiotherapists and was inspired by neck exercise instructions, as described in the appendix of Tunwattanapong et al. [17]. We designed the audio and visual interface iteratively in a participatory approach, emphasizing the aesthetic aspects of the user experience.

#### 3.2.1. Hardware

In the final prototype, the Arduino UNO board was substituted with the LightBlue Bean board [35]. The LightBlue Bean uses the AtMega328p Arduino Microcontroller and the LBM313 Bluetooth module. This is a small-sized development board that is battery-powered and uses Bluetooth Low Energy for wireless data transmission.

**Figure 3:**
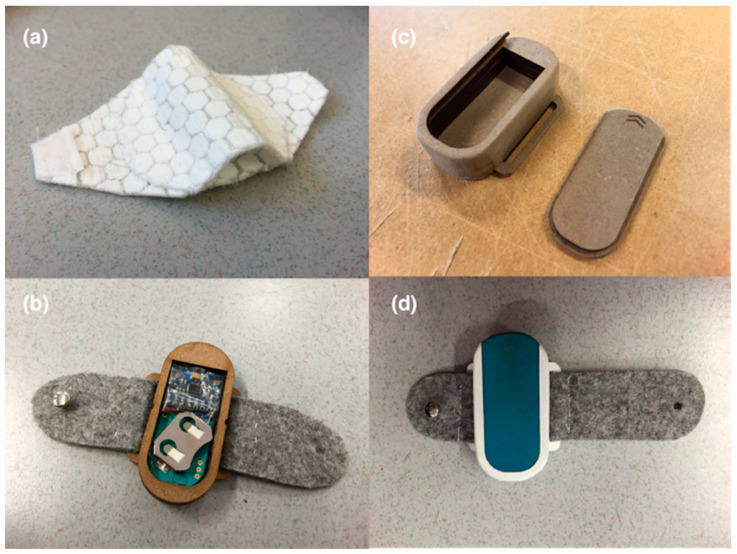
Successive iterations in product design: (**a**) initial fabric sensor holder; (**b**) laser-cut wooden casing of sensors with felt bands; (**c**) the construction design of the case which can be opened by sliding the lid; (**d**) final prototype wooden casing with adjusted lengths of felt bands.

The tracking device has been designed as an adjustable band, which can be easily mounted on the top of the headset. The first prototype was made of fabric (Figure 3a) so it was too easily deformed and hard to keep at the desired position on the headset. To resolve this problem, a wooden casing with two felt bands was made to contain and fix the electronics on the headphone (Figure 3b). However, the hard metal button caused discomfort, so the strap was adjusted so that the metal button stays at the side of headphone to avoid contact with head (Figure 3d). Figure 3c shows the construction design of the casing which can be opened by sliding the lid. The prototype weighs 32 gr and the dimensions of the casing are (3 cm × 5.8 cm × 1.2 cm).

#### 3.2.2. Software

The Neckio application was developed in Processing, which is compatible with the LightBlue Bean board [35]. The software runs in the background of the computer and can be brought to the foreground at any time. Data from the tracking device is continuously transmitted to the Neckio application via a Bluetooth connection at a frequency of 200 Hz. So far, the application is only available on macOS. The Neckio application supports four functions:

- Exercise reminder
- Interactive neck exercise
- Work duration tracking
- Posture tracking.

**Figure 4:**
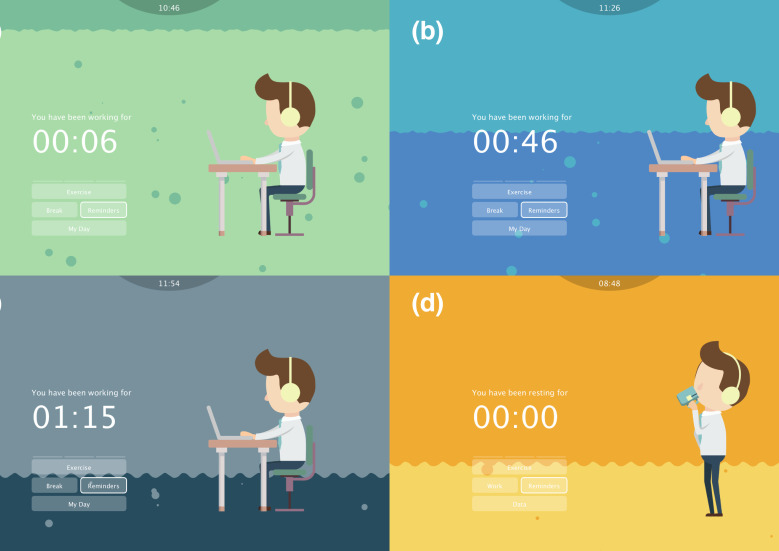
Overview of the home page in different states: working less than 30 min since (**a**) the last break; (**b**) 30–60 min since the last break; (**c**) over 1 h since the last break; and (**d**) break.

The home page displays the user’s current state (work/rest) and its duration, encouraging at least one break per hour. The visual background was inspired by the ebb and tide (Figure 4). The user can switch states by clicking the button of “Work” or “Break”. The water in the background drains away slowly while user keeps working to illustrate the time elapsed since the last break. The colour of the water changes from green (0–30 min) to blue (30–60 min), and then to grey (more than 60 min). An audio notification playing a sound of bubbles is activated when the duration of a continuous work episode exceeds an hour. When switched to “Break”, the water becomes yellow and its level rises again. It takes 20 min at most to reach the starting level.

**Figure 5:**
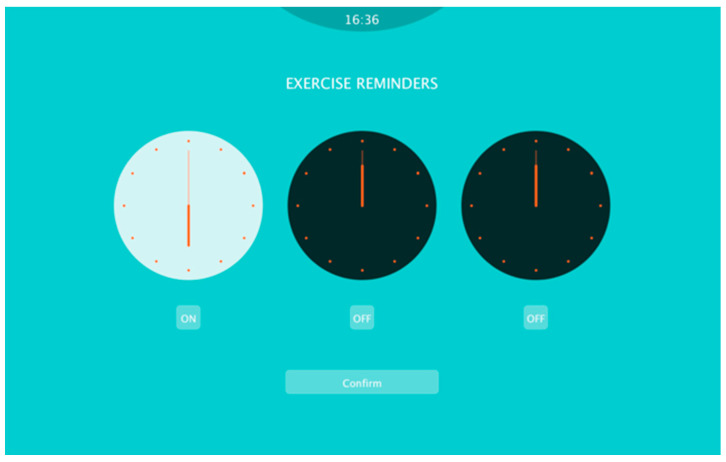
To personalize the reminder settings, the user can rotate the hands of the clock to set reminder options for the timing of three alarms during the working date.

**Figure 6:**
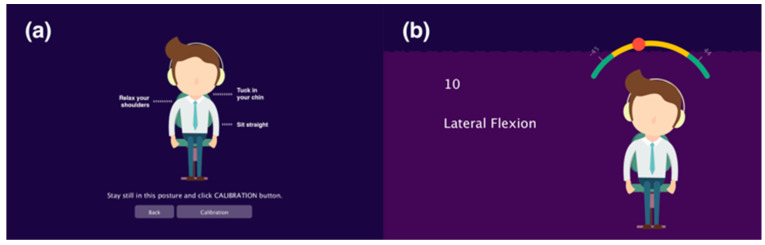
User interface of (**a**) device calibration and (**b**) exercise.

In the “Reminder” page (Figure 5), the user can schedule neck exercises and set reminders by rotating the hand of the clocks and pushing the switch button. When the reminder goes off, soft meditation music plays smoothly to remind the user to exercise. Furthermore, to ensure correct operation, the device needs to be calibrated by setting the angulation detected in a straight sitting posture to zero. Before the exercise starts, the starting posture is introduced and the user is required to calibrate the device in this posture (Figure 6a).

**Figure 7:**
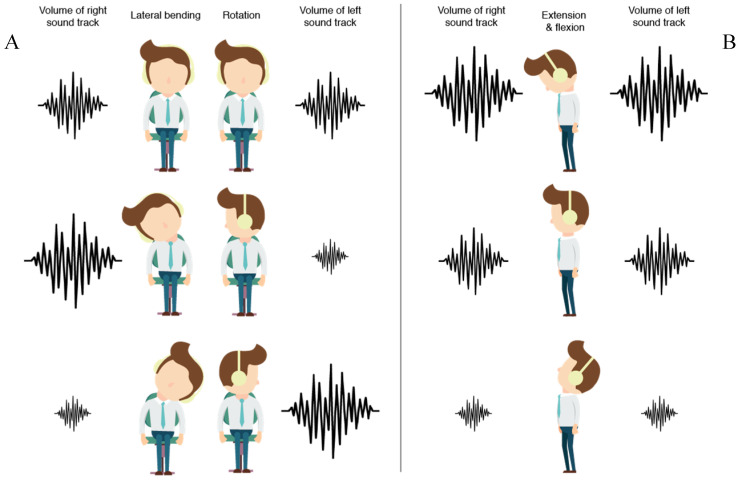
Audio feedforward. (**A**) Different volumes of left and right sound tracks indicate the direction of lateral bending and rotation exercises. (**B**) The total volume of audio indicates the direction of the extension & flexion exercises.

After calibration, the user will be guided to perform the three exercises that were mentioned in the previous section: lateral bending, rotation and extension and flexion (Figure 6b). Exercise guidance is given in both the visual and the audio modalities. Character animations are displayed to illustrate the exercise movements. The desired direction of the head is indicated by manipulating the relative sound volume on the left and right, while the speed and the moment to change direction are expressed through adjusting the total volume of the audio (Figure 7).

The visual feedback of the head movements is displayed in a similar way as in the previous iteration: a circle moves along a circular scale bar to indicate the rotation angle on the corresponding axis, which is different for the various exercises. To provide a clear visualization of exercise performance, the normal range of motion is highlighted on the scale bar. When reaching the normal range, the moving circle will turn green to indicate the healthy condition.

**Figure 8:**
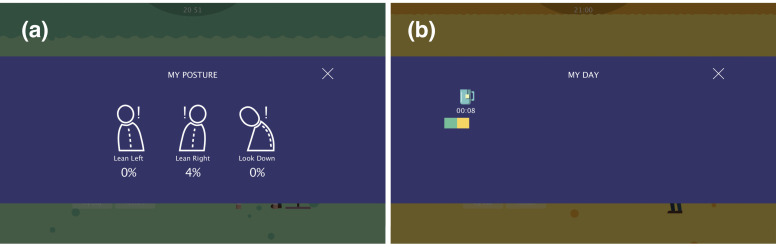
User interface (**a**) “Posture” page (**a**) and (**b**) “My Day”.

**Figure 9:**
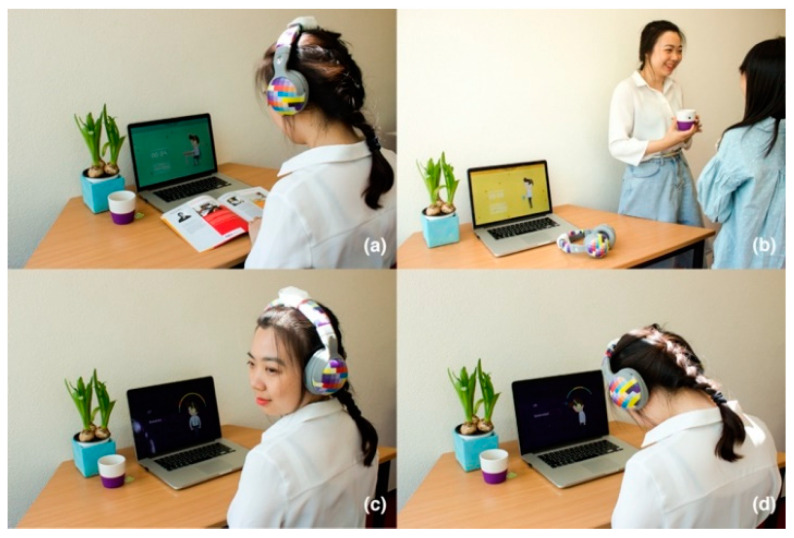
Modes of Neckio use (**a**) tracking mode during work; (**b**) rest; (**c**) exercise: rotation; (**d**) exercise: extension and flexion.

During computer-related work, the sitting posture is tracked by the device. The percentages of time a user spends in a certain position is calculated and shown in the “Posture” page (Figure 8a), which aims to raise users’ awareness of their own habitual sitting posture. “My Day” page (Figure 8b) presents an overview of daily activities during work hours, which includes working, resting and exercising. Figure 9 illustrates the interactions between the user and the Neckio system in context.

## 4. User Test

As an initial evaluation of the design concept, we carried out a structured user test that focused on how users experience the use of Neckio and how motivated they are to use it. This test was conducted in field conditions with participants trying out the system on their own workstation and in their actual working environment (library, open office, etc.). The methods used and the results are presented below.

### 4.1. Methods

#### 4.1.1. Participants

The user test was carried out with a convenience sample of young adults. The criterion for inclusion was to be working for roughly six hours per day on a computer and to have no neck pain or injury. A total of ten students with ages 18–30 participated.

#### 4.1.2. Measures

We used the User Experience Questionnaire [36] as a quantitative measure of the user experience, because it takes a few minutes to fill in, it allows a holistic assessment of the emergent user experience in 6 subscales and, importantly, because it allows benchmarking a prototype against a large set of previously tested products. The user experience is rated by 26 seven-point Likert scales organized in 6 groups corresponding to the following dimensions of user experience: Attractiveness, Perspicuity, Efficiency, Dependability, Stimulation, and Novelty.

The Intrinsic Motivation Inventory [37] is a multi-dimensional scale for assessing the intrinsic motivation and self-regulation of participants for a target activity, which has very good psychometric properties [38]. We used an adapted version consisting of 26 items (also 7-point Likert scales) that are grouped in 6 dimensions: interest/enjoyment, perceived competence, effort, value/usefulness, felt pressure and tension, and perceived choice while performing a given activity. The interest/enjoyment subscale is considered the core self-report measure for intrinsic motivation.

Finally, we interviewed users regarding the wearability of the Neckio band, the social deviance it might cause, the overall exercise experience and its overall potential usefulness for them.

#### 4.1.3. Procedure

Participants tested the system in their actual working environments while doing their own work. Participants were introduced to the purpose of the study and provided informed consent. Then, the Neckio application was installed on their laptops and connected to the Neckio band (tracking device). The test lasted two hours. First, participants initialized the system and calibrated it and then they were asked to perform three tasks in order to experience the different aspects of Neckio: (a) setting the duration tracking function shifting states between “work” and “break” according to their actual work pace, (b) setting up an exercise reminder, (c) going on with their work and carrying out the neck exercises when the system would ask them to. At the end of the session, they filled in the two questionnaires and were interviewed regarding their experience.

### 4.2. Results

#### 4.2.1. User Experience Questionnaire (UEQ)

**Figure 10:**
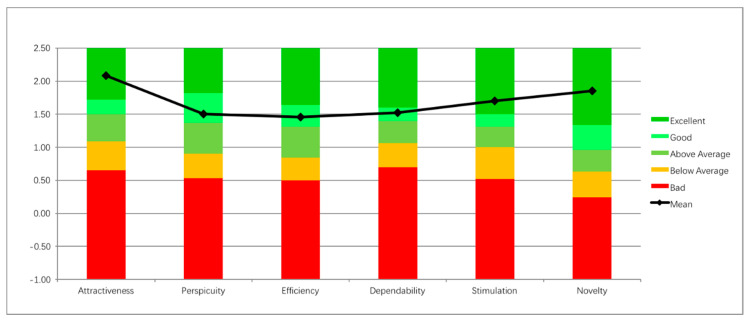
Benchmarking with other products tested with the UEQ questionnaire provides excellent or positive results along all subscales.

Table 1 and Figure 10 show the average scores of each scale and the confidence intervals from the UEQ, which have been averaged and mapped on a scale ranging from −3 (lowest) to +3 (highest). The standard interpretation [39] of the UEQ scale is that values between −0.8 and 0.8 represent a neutral evaluation of the corresponding scale, values >0.8 represent a positive evaluation and values < −0.8 represent a negative evaluation. As can be seen in Table 1, the system was evaluated positively on all scales.

The UEQ [13] offers a benchmark, based on data collected from 163 product evaluations with the UEQ (with a total of 4818 participants in all evaluations). The benchmark classifies a product into five categories (per scale) ranging from bad to excellent (see [40]). Neckio is benchmarked as excellent (meaning that it is in the top 10% among the products evaluated with the UEQ) in the Attractiveness, Stimulation and Novelty dimensions. It is benchmarked as Good (meaning 10% of the results in the benchmark data set are better and 75% of the results are worse) in the remaining subscales. The comparison to the benchmark set is illustrated in Figure 10. These results are very satisfactory as the benchmark includes mainly mature commercial products and the expectations on UEQ are continuously shifting and increasing as years go by [41].

#### 4.2.2. Intrinsic Motivation Inventory (IMI)

The average user scores obtained for the intrinsic motivation inventory are averaged per sub-scale for all participants, as shown in Table 2. The interest/enjoyment sub-scale was rated very highly, indicating that participants are strongly motivated to use Neckio. The value/usefulness scale also received a high score, which suggests that the Neckio system is found useful and valuable for one’s health. These positive evaluations would suggest that users can internalize and become self-regulating with respect to using the system (e.g., see [41]). The perceived choice and perceived competence concepts are theorized to be positive predictors of intrinsic motivation, and pressure/tension is theorized to be a negative predictor of intrinsic motivation [37]. As Table 2 shows, Neckio was considered to be easy to master (perceived competence), and it leaves space and freedom for users to choose what they want to do without coercing them (perceived choice). Effort/importance is a separate variable to measure the perceived effort user spends on using the system. According to this result, participants found the system relatively effortless to use.

#### 4.2.3. Interview Results

A short interview was held to examine how participants experienced the wearability of the device, the social deviation it causes, and the overall exercise experience and perceived value. All participants were positive regarding the wearability of the Neckio band, as they did not feel the band after it had been mounted on their headset. Two of the ten participants expressed a slight apprehension for performing neck exercises at their workplace, which are relatively crowded. One participant described the Neckio exercise as relaxing and easy to learn. “*Neckio is very useful to use at my work pace. While doing exercises, I did feel my neck was kind of stiff...The exercise is quite relaxing, and the audio effect is easy to follow.*” Another participant commented that the visual feedback was motivational for carrying out the exercises in place. “*Exercising with Neckio makes me more aware of my neck movement range… I tried very hard to reach the green part.*” In terms of its perceived value, Neckio was generally considered to be motivational for taking regular breaks and performing exercises. Unexpectedly, one participant even thought it might improve work efficiency through tracking work duration. “*I think Neckio boosts my work efficiency, because I got better track of my working time.*”

Participants also made suggestions for improvement. For example, they suggested enhancing audio feedback for the head position so that the exercise performance can be heard when the user is not facing the screen. “*I can’t see the feedback while doing exercise like rotation. It may be better to have audio feedback too, so I can hear how I am doing without looking at the screen.*” It was suggested to display the basic information in a floating window, so that it could be available in a peripheral way during work. Furthermore, it was suggested that enhancing the exercises with gamification can make them more engaging and fun.

## 5. Discussion and Conclusions

The aim of this research has been to (1) collect insights into the needs of the target group (computer users at risk for development of musculoskeletal problems), (2) to describe the prototyping of Neckio based on these insights, and (3) to evaluate the resulting system with a user test conducted in situ.

We surveyed a sample of 130 relatively young computer workers who spend more than 6 h per day in front of a computer, which is known to result in work-related upper limb disorders in all body regions [6]. The survey showed that these participants can mainly be supported by increasing their ability to carry out the exercises in terms of reminding them to do them, instructing them how to do the exercises and minimizing social deviance (not making them appear strange while they do them).

Neckio is a neck care system that was designed in an iterative design process that targets computer workers who use headsets in open office environments. These headsets are currently used to isolate their users from distractions and are used in Neckio to mount a sensor for monitoring head position and to deliver directional audio feedback. An inertial sensor is connected to an application that provides reminders and instructions to users, while audio feedback is used to guide the direction and pace of exercises, which are gently performed by leaning the head sideways or rotating the head left and right. The resulting exercises can be done without attracting negative attention and are an intended to be aesthetically pleasant.

The results confirm that that users experienced Neckio positively and were motivated to perform the exercises. Moreover, comments obtained through interviews suggest that the device is easy to wear and most users do not feel apprehensive about doing the exercises in a shared office space. While these results are promising, we do not yet know whether Neckio can be effective in preventing neck pain. To evaluate this, follow-up research is needed, which should involve a lager sample of participants, taking part in a randomized controlled trial and examining the use over at least 4 weeks, which has been shown in the past to result in improvements in the neck condition. As work-related sustained muscle activity has been related to neck pain [42], it would be interesting to evaluate whether and to what extent an intervention with Neckio could influence this relationship.

This study has emphasized the interaction design that can contribute towards a positive behavior change, to remind computer workers to train and to guide them in doing so. Future work could further elaborate and improve upon the technical performance of the system, especially focusing on the sensor accuracy and hysteresis, especially after repeated movements.

Further developments regarding the interaction design could enhance the audio feedback to provide some redundancy with the information currently provided on the screen. This can help reduce the need for looking at the display and help in exercises that involve turning the head. In order to make the transition from a research prototype to a product, the LightBlue Bean can be replaced with smaller purpose-specific and energy-saving electronics that integrate only the necessary functionality, namely the low-energy Bluetooth, the sensors and a rechargeable battery. The current Neckio prototype is very light (32 g), which does not bring risks for straining the user’s neck excessively. Further development of this research prototype toward a product will use different manufacturing techniques (e.g., printed circuits instead of a prototyping board, molding instead of wooden casing), which can dramatically reduce its dimensions and weight.

Finally, the current project has examined neck health for computer users. Sustained head-down use of handheld devices (especially smartphones) can also cause of neck pain [43], and it would be interesting to develop a coherent and holistic solution that addresses both contexts.

## Figures and Tables

**Table 1:** Ratings of the participants on the user experience questionnaire (UEQ) scale. One sample of *t*-tests compared the mean scores to the neutral point of the scale. Statistically significant items are marked with an *.

Scale	Mean	SD	*p* from One Sample *t*-Test
Attractiveness	2.08	0.708	0.439 *
Perspicuity	1.50	0.920	0.570
Efficiency	1.46	0.816	0.506
Dependability	1.53	0.768	0.476 *
Stimulation	1.70	0.715	0.443 *
Novelty	1.85	0.592	1.483

**Table 2:** Scores of participants per subscale of the intrinsic motivation inventory. *p*-values for comparison to the middle point of the scale and 95% confidence intervals are shown.

Subscale	Mean	Std. Dev.	*p*	95% Conf. Interval
Effort/importance	4.23	0.93	0.58	[3.65–4.80]
Interest/Enjoyment	6.02	0.44	0.27	[5.74–6.29]
Perceived Competence	5.90	0.87	0.54	[5.36–6.44]
Perceived Choice	5.50	0.58	0.36	[5.14,6.44]
Pressure/Tension	2.00	0.53	0.33	[1.67–2.33]
Value Usefulness	5.90	0.99	0.61	[5.29–6.51]
